# *RUNX2* tandem repeats and the evolution of facial length in placental mammals

**DOI:** 10.1186/1471-2148-12-103

**Published:** 2012-06-28

**Authors:** Marie A Pointer, Jason M Kamilar, Vera Warmuth, Stephen G B Chester, Frédéric Delsuc, Nicholas I Mundy, Robert J Asher, Brenda J Bradley

**Affiliations:** 1 Department of Zoology, University of Cambridge, Cambridge, CB2 3EJ, UK; 2 Department of Anthropology, Yale University, New Haven, Connecticut 06511, USA; 3 Department of Zoology, University of Oxford, Oxford, OX1 3PS, UK; 4 Department of Anatomy, Midwestern University, Glendale, Arizona 85308, USA; 5 School of Human Evolution and Social Change, Arizona State University, Tempe, Arizona 85287, USA; 6 Institut des Sciences de l'Evolution, UMR5554-CNRS-IRD, Université Montpellier II, Montpellier, France

**Keywords:** Mammalian evolution, Prognathism, Molecular evolution, Primates, Afrotheria, Xenarthra, Morphology

## Abstract

**Background:**

When simple sequence repeats are integrated into functional genes, they can potentially act as evolutionary ‘tuning knobs’, supplying abundant genetic variation with minimal risk of pleiotropic deleterious effects. The genetic basis of variation in facial shape and length represents a possible example of this phenomenon. Runt-related transcription factor 2 (RUNX2), which is involved in osteoblast differentiation, contains a functionally-important tandem repeat of glutamine and alanine amino acids. The ratio of glutamines to alanines (the QA ratio) in this protein seemingly influences the regulation of bone development. Notably, in domestic breeds of dog, and in carnivorans in general, the ratio of glutamines to alanines is strongly correlated with facial length.

**Results:**

In this study we examine whether this correlation holds true across placental mammals, particularly those mammals for which facial length is highly variable and related to adaptive behavior and lifestyle (e.g., primates, afrotherians, xenarthrans). We obtained relative facial length measurements and RUNX2 sequences for 41 mammalian species representing 12 orders. Using both a phylogenetic generalized least squares model and a recently-developed Bayesian comparative method, we tested for a correlation between genetic and morphometric data while controlling for phylogeny, evolutionary rates, and divergence times. Non-carnivoran taxa generally had substantially lower glutamine-alanine ratios than carnivorans (primates and xenarthrans with means of 1.34 and 1.25, respectively, compared to a mean of 3.1 for carnivorans), and we found no correlation between *RUNX2* sequence and face length across placental mammals.

**Conclusions:**

Results of our diverse comparative phylogenetic analyses indicate that QA ratio does not consistently correlate with face length across the 41 mammalian taxa considered. Thus, although RUNX2 might function as a ‘tuning knob’ modifying face length in carnivorans, this relationship is not conserved across mammals in general.

## Background

Work on the molecular basis of animal adaptation is providing ample evidence that small genetic changes can underlie striking phenotypic differences, both within and between species [1-4]. Of recent particular interest is the role of short tandemly repeating DNA elements (i.e. microsatellites) as evolutionary ‘tuning knobs’ [5]. Microsatellites are mutation-prone and often show substantial length variation in repeat number [6]. These repeats are common in mammalian genomes, often occurring within coding exons, potentially translating into expansions and contractions of poly-amino acid stretches [7,8]. Moreover, proteins containing such repeats are often involved in development [9,10]. Thus, the function of a protein can be easily modified via alterations in repeat tract length [8,10] and such modifications may have less drastic pleiotropic effects than other types of mutation [4].

An example of this is the correlation between specific tandem repeats and variation in midfacial length (i.e., the degree of prognathism, or the jutting of the face and jaw) in carnivorans [11-13]. Variation in this striking morphological trait appears to be causatively associated with variation in coding sequence repeats within the gene *RUNX2* (Runt-related transcription factor 2).

*RUNX2* (also known as *Cbfa1*; Entrez Gene ID 860) codes for a transcription factor that plays an important role in mediating osteoblast differentiation and activity [14]. Its function is critical for building and repairing bone. The activity of RUNX2 is mediated by a few functional domains, one of which consists of a stretch of glutamines (abbreviated Q) followed by a stretch of alanines (A) (reviewed in [15]). For RUNX2, the ratio of glutamines to alanines (QA ratio) appears to be positively correlated with the transcriptional activity of the protein [13,16].

*RUNX2* mutations in humans cause the skeletal disease cleidocranial dysplasia (MIM 119600) and have been associated with a shortening or protruding of the face and other skeletodental pathologies [17,18], some of which correspond to changes in certain clades of nonhuman mammals [19]. In general, work on *RUNX2* in humans and mice indicates that when *RUNX2* is up-regulated, bone development is accelerated [20]. Interestingly, in comparisons of the modern human and Neanderthal genomes, *RUNX2* is specifically highlighted by Green et al. [21] as one of few genes showing a genetic signature of a selective sweep in the modern human lineage. Assuming the Neanderthal sequence data are valid, this would suggest a potentially important role for *RUNX2* in human-specific cranial/skeletal features.

The QA ratio of RUNX2 is correlated with facial length across breeds of dog (92 breeds of *Canis lupus familiaris*; see [11] and across carnivorans in general (30 species; [13]). Specifically, dog breeds and other carnivoran species with a high QA ratio tend to have a relatively long rostrum or "face". Furthermore, Sears et al. [13] noted that the correlation was stronger among dogs, bears, raccoons and their relatives (i.e., caniforms) than among cats, civets, and mongooses (i.e., feliforms), related to the fact that the rostrum in caniforms shows more positive allometry during growth than in feliforms. That is, compared to a cat, a dog shows more growth in the face relative to the braincase during its ontogeny.

The possibility that this correlation represents a general mechanism responsible for at least some aspects of skull growth across non-carnivoran mammals has not yet been thoroughly investigated. Facial shape and length are highly variable in many mammalian clades, including afrotherians (e.g., tenrecs), xenarthrans (e.g., anteaters) and euarchontoglires (e.g., primates). Morphometrically, the skull shows a tremendous amount of continuous variation across mammals, an observation which lends itself to the metaphor of the "tuning knob" as a genetic basis behind this variation [5,22]. *RUNX2*, specifically the QA ratio, is thus a good candidate for examining the genetic basis of variation in skull proportions across mammals.

**Figure 1:**
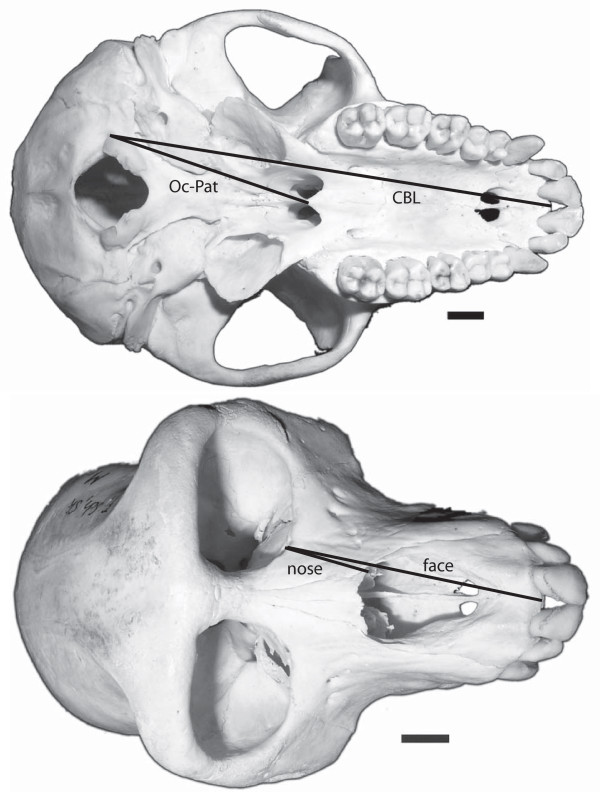
**Cranial measurements taken in this study.** Macaque cranium in (A) ventral and (B) rostral views. CBL = distance from lateral-most point of occipital condyle to anterior premaxilla; Oc-Pat = distance from lateral-most point of occipital condyle to caudal margin of palate; Nose = anteroventral margin of orbit at lacrimal foramen to anterolateral margin of nasal bone; Face = anteroventral margin of orbit at lacrimal foramen to anterior premaxilla (specimen: ZMB 70003). Scale bar = 10mm.

**Figure 2:**
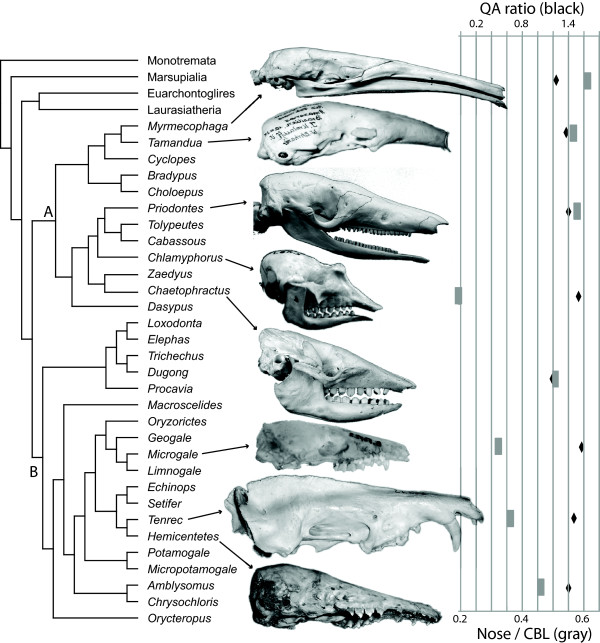
**Phylogeny of xenarthrans and afrotherians illustrating lack of correlation between QA ratio and face length.** The phylogeny of xenarthrans (**A**, after [24]; position of *Chlamyphorus* after [25]) and afrotherians (**B**, after [26,27]) is given with corresponding facial proportions and QA ratios in selected species using graph at right. Gray boxes represent ratio face-to-skull length (bottom scale; higher values indicate a longer face); black diamonds represent ratio of glutamine to alanine in RUNX2 binding site (top scale). Note relative lack of correlation between large (e.g., *Myrmecophaga*) vs. short- (e.g., *Chlamyphorus*) faced species. Specimens shown (not to scale) are *Myrmecophaga tridactyla* (AMNH 1873), *Tamandua tetradactyla* (ZMB 38396, image reversed), *Priodontes sp*. (AMNH 1871), *Chaetophractus villosus* (AMNH 240), *Chlamyphorus truncatus* (AMNH 5487), *Microgale dobsoni* (UMZC 5458-B), *Tenrec ecaudatus* ZMB 90377), and *Hemicentetes semispinosus* (ZMB 5007).

**Figure 3:**
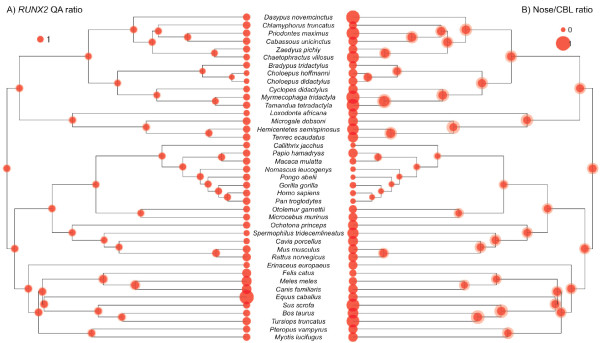
**Joint Bayesian reconstructions of RUNX2 QA ratios and relative nose length in 41 placental mammals.** Reconstructions for RUNX2 QA ratios are given on the left (**A**), while reconstructions for relative nose length (Nose/CBL ratio) are on the right (**B**). Dark and light shaded disks at nodes indicate 95% credibility intervals of inferred ancestral ratios.

Here we assess the extent to which the correlation between skull shape and RUNX2 QA (Figure 1, Table 1) ratio holds true across a variety of non-carnivoran placental mammals. In particular, we focus on xenarthrans, afrotherians, and primates as these orders show marked variations in face length, which are thought to be adaptively associated with diet and ecology [23], and for these orders we have access to morphometric and genetic data. In analyzing data for 41 placental mammal species representing 12 orders (Table 1), we find no correlation between RUNX2 sequence and face length (Figures 2, and 3; Tables 2, and 3) across our sample as a whole.

**Table 1:** Mammalian taxa examined in this study, RUNX2 QA ratios, sources of sequence data, and face-length measures

**Order**	**Species**	**Common name**	**RUNX2 sequence source**	**Q**	**A**	**QA ratio**	Nose/Oc-Pat*	Nose/CBL*	Face/Oc-Pat*	Face/CBL*
Euarchontoglires										
Primates	*Callithrix jacchus*	Common marmoset	ENSCJAG00000012153	21	17	1.24	0.18	0.11	0.41	0.25
	*Gorilla gorilla*	Western gorilla	ENSGGOG00000012890	22	17	1.29	0.33	0.16	0.98	0.47
	*Homo sapiens*	Human	ENSG00000124813	23	17	1.35	0.26	0.13	0.88	0.46
	*Macaca mulatta*	Rhesus macaque	ENSMMUG00000019819	24	17	1.41	0.37	0.17	0.99	0.47
	*Microcebus murinus*	Gray mouse lemur	ENSMICG00000012251	20 (14Q H 6Q)	16	1.25	0.55	0.30	0.64	0.35
	*Nomascus leucogenys*	White-cheeked gibbon	BLAST: wgs read 1906216859	23	17	1.35	0.21	0.11	0.58	0.31
	*Otolemur garnettii*	Small-eared galago	ENSOGAG00000003900	21	17	1.24	0.45	0.25	0.57	0.31
	*Pan troglodytes*	Chimpanzee	ENSPTRG00000018228	25	17	1.47	0.24	0.12	0.98	0.47
	*Papio hamadryas*	Hamadryas baboon	BLAST: wgs read 1986882716	24	17	1.41	0.85	0.34	1.57	0.63
	*Pongo abelii*	Sumatran orangutan	ENSPPYG00000016664	23	17	1.35	0.19	0.10	0.89	0.47
Rodentia	*Cavia porcellus*	Guinea-pig	BLAST: wgs read 1611098943	18	17	1.06	0.82	0.39	1.01	0.48
	*Mus musculus*	House mouse	ENSMUSG00000039153	29	18	1.61	0.75	0.34	0.81	0.36
	*Rattus norvegicus*	Brown rat	ENSRNOG00000020193	31	17	1.82	0.82	0.34	0.97	0.41
	*Spermophilus tridecemlineatus*	Thirteen-lined ground squirrel	ENSSTOG00000010163	16	16	1.00	1.18	0.47	1.04	0.42
Lagomorpha	*Ochotona princeps*	American pika	ENSOPRG00000002943	23	17	1.35	0.47	0.39	0.48	0.40
Laurasiatheria										
Cetartiodactyla	*Bos taurus*	Cow	BLAST: XM_002697263	21	16	1.31	1.03	0.44	1.45	0.62
	*Sus scrofa*	Pig	ENSSSCG00000001710	22	17	1.29	2.05	0.64	2.41	0.75
	*Tursiops truncatus*	Bottlenose dolphin	ENSTTRG00000006943	23	13	1.77	1.81	0.63	1.88	0.65
Perissodactyla	*Equus caballus*	Horse	ENSECAG00000020875	16	3	5.33	0.67	0.33	1.29	0.64
Carnivora	*Canis familiaris (2)*	Dog (Breed: huskey)	this study	20	8	2.50	0.75	0.35	1.04	0.48
	*Felis catus*	Cat	this study	21	10	2.10	0.34	0.20	0.50	0.29
	*Meles meles*	Badger	this study	19	7	2.71	0.59	0.26	0.87	0.38
Chiroptera	*Myotis lucifugus*	Microbat/Little brown bat	BLAST: wgs read 992289586	20	14	1.43	0.70	0.34	0.99	0.48
	*Pteropus vampyrus*	Megabat/Large flying fox	ENSPVAG00000001720	22	16	1.38	0.72	0.32	0.89	0.40
Erinaceomorpha	*Erinaceus europaeus*	European hedgehog	ENSEEUG00000009243	14	13	1.08	0.69	0.30	0.85	0.37
Afrotheria										
Proboscidea	*Loxodonta africana*	African bush elephant	BLAST: wgs read 468964616	18	17	1.06	0.93	0.33	1.43	0.50
Tenrecomorpha	*Hemicentetes semispinosus*	Streaked tenrec	this study	21	15 (14A V 1A)	1.40	1.06	0.51	0.90	0.43
	*Microgale dobsoni*	Dobson's shrew tenrec	this study	22	14 (12A V 2A)	1.57	0.72	0.37	0.81	0.42
	*Tenrec ecaudatus*	Tailless tenrec	this study	22	15 (13A V 2A)	1.47	1.01	0.41	1.35	0.55
Xenarthra										
Pilosa	*Bradypus tridactylus*	Pale-throated three-toed sloth	this study	16	13	1.23	0.39	0.24	0.41	0.25
	*Choloepus didactylus (3)*	Linnaeus's two-toed sloth	this study	14	17	0.82	0.54	0.30	0.59	0.33
	*Choloepus hoffmanni*	Hoffman's two-toed sloth	this study	14	16	0.88	0.55	0.30	0.62	0.33
	*Cyclopes didactylus*	Silky anteater	this study	18	15	1.20	0.62	0.34	0.62	0.34
	*Myrmecophaga tridactyla (2)*	Giant anteater	this study	21	18 or 16 (14A V 2A)	1.24	7.46	0.66	7.29	0.65
	*Tamandua tetradactyla (3)*	Southern tamandua	this study	21-22	15-16 (13-14A V 2A)	1.37	0.98	0.62	0.98	0.61
Cingulata	*Cabassous unicinctus*	Southern naked-tailed armadillo	this study	20	14 (12A V 2A)	1.43	0.86	0.32	1.35	0.50
	*Chaetophractus villosus*	Large hairy armadillo	this study	19	16	1.19	1.47	0.56	1.45	0.55
	*Chlamyphorus truncatus*	Pink fairy armadillo	this study	23	15 (13A V 2A)	1.53	0.62	0.24	1.46	0.57
	*Dasypus novemcinctus*	Nine-banded armadillo	BLAST: wgs read 1959731662	22	16 (14A V 2A)	1.38	2.55	0.71	2.33	0.65
	*Priodontes maximus*	Giant armadillo	this study	21	15 (13A V 2A)	1.40	1.96	0.63	1.78	0.57
	*Zaedyus pichiy*	Dwarf armadillo	this study	21	16 (14A V 2A)	1.31	0.72	0.29	1.34	0.54

*See Figure 1 for measurement details.

When greater than one, the number of individuals is given in parentheses.

**Table 2:** Results of phylogenetic generalized least squares models examining the potential relationship between RUNX2 QA ratios and relative face-length and nose-length in mammals

**Model**	**r**^**2**^	**Estimate**	**p**	**Pagel's lambda**
QA ratio vs. Face/Oc-Pat^1^	0.000	-0.027	0.989	0.000
QA ratio vs. Face/CBL^1^	0.000	-0.018	0.990	1.000
QA ratio vs. Nose/Oc-Pat^1^	0.012	-0.246	0.622	0.615
QA ratio vs. Nose/CBL^1^	0.015	-0.183	0.553	0.817

^1^All variables were natural log transformed before analysis. See Figure 1 for measurement details.

**Table 3:** **Results of Bayesian comparative analyses as implemented in the CoEvol program** [[28]]

	**Taxa**	**Phylogenetic control dataset**	**R2**	**Correlation coefficient**	**Posterior probability**
Sears et al. data [13]	30 carnivores	MT-CYTB	0.0801	0.28	0.95*
QA ratio vs. Face/Oc-Pat^1^	41 placentals	VWF exon 28	0.0001	0.01	0.51^ns^
QA ratio vs. Face/CBL^1^	41 placentals	VWF exon 28	0.0094	0.10	0.73^ns^
QA ratio vs. Nose/Oc-Pat^1^	41 placentals	VWF exon 28	0.0110	0.10	0.25^ns^
QA ratio vs. Nose/CBL^1^	41 placentals	VWF exon 28	0.0102	0.10	0.26^ns^

^1^See Figure 1 for measurement details; * Statistically significant; ^ns^ Not significant.

## Results

### RUNX2 tandem repeat ratios

The translated amino acid sequences for the 41 species are provided in the Supplementary Material ( Additional File 1). The RUNX2 amino acid sequences flanking the tandem repeat region were generally conserved across taxa providing evidence that orthologous rather than paralogous sequences were successfully retrieved. In support of this, all publicly-available mammalian genomes contain a single annotated *RUNX2* locus, suggesting that this gene has 1:1 orthology across mammals and has not been subject to duplication. QA ratios calculated from the translated sequences range from 0.82 (*Choloepus didactylus*, Xenarthra) to 5.33 (*Equus caballus*, Perissodactyla, but see below) (Table 1). With the exception of *Equus*, these QA ratios were generally lower than those of carnivorans [13]. In their analysis of Carnivora, Sears et al. [13] report a lowest value of 1.5 for the QA ratio in *Potos flavus*, which is close to the range of domesticated dog breeds (approximately 1.2 – 1.45; based on Figure 2B in [11]). Glutamine and alanine amino acid counts for non-carnivoran mammals (glutamines: 14-29, alanines: 13-18) were not markedly different than those of domestic dog breeds (glutamines: 18-20; alanines: 12-17), although carnivorans are on the low end of alanine counts (Table 1). Across the mammals included in our dataset, the majority of the QA ratios lie between 1 and 2 (34 out of 41 species analyzed). Xenarthrans had the lowest variation in QA ratio across sampled species (variance = 0.067) and laurasiatherians had the highest (variance = 1.60). We make such taxonomic comparisons tentatively, as species within clades are not equally distant to each other and the species sampled here represent only a subset of the extant diversity for each clade.

Across the 41 mammals tested, both the polyQ and polyA are variable, but the polyQ has a greater range of values than the polyA repeat (polyQ ranges from 10-31 repeats, polyA ranges from 3-19 repeats) ( Additional File 1). Also, intraspecific variation was detected in two (*Myrmecophaga tridactyla*, the giant anteater, and *Tamandua tetradactyla*, the southern tamandua) of the four species where multiple individuals were sequenced (Table 1).

Although *RUNX2* is generally conserved across mammals, three of the *RUNX2* sequences obtained via genome browsers (three-lined squirrel, bottlenose dolphin and horse) contain distinctive amino acid changes ( Additional File 1). We cannot conclusively exclude the possibility of errors in the online genome sequence annotations, and this is of particular concern for the horse where there is extensive divergence in the 3’ flanking sequence. The unusually high QA ratio of 5.33 for *Equus caballus* is mostly due to the highly shortened poly A sequence (3 repeats compared to 7-17 repeats for other laurasiatherians), and, thus, this result should be viewed with caution. However, there is no a priori reason to suspect sequence or annotation errors for the three-lined squirrel or bottlenose dolphin; in these cases the sequences flanking the unusual amino acids are highly conserved, as are other mammalian sequences. We also note that the flanking sequences for some of the novel species (*Dasypus*, *Hemicentetes*) are short, but in these cases we can confirm the sequence from original trace files. The removal of flanking sequence was done when an ambiguous base pair was present in the sequence trace file. Sequences were only included in both the alignment and further analysis if the QA region of the sequence contained no ambiguous trace file calls; thus the QA ratio is likely to be correct in these cases, even though the flanking sequence was partially truncated.

The polyA repeat was often interrupted by a single valine ( Additional File 1). In fact, the presence of an interrupting amino acid has an interesting phylogenetic distribution (and intraspecific variation in *Myrmecophaga*), as most afrotherians and xenarthrans exhibit an interrupted polyA sequence but laurasiatherians or euarchontoglires do not. Regarding the polyQ repeat, only *Microcebus murinus*, the grey mouse lemur, has an interrupted glutamine sequence (by histidine; Table 1). In further analyses (see below), we considered both the total QA ratio (ignoring any interrupting amino acids) and the QA ratio for uninterrupted sequences only. For example, we scored the total QA ratio for the mouse lemur as 1.25 (20Q : 16A) in the initial analysis, but we also ran the analyses with a mouse lemur QA ratio of 0.38 (6Q : 16A) (Table 1). Whether we included total QA ratios or uninterrupted QA ratios, the direction and significance of the correlation results did not change (Table 2).

Since the goal of this study is to examine whether the pattern Sears et al. [13] describe for carnivorans holds for mammals in general, we took morphological measurements comparable to those described in Sears et al. [13] (Figure 1). After adjusting measurements relative to body size by dividing by proxy measures of overall size (following [13]; CBL, condylobasal length; and Oc-Pat, distance from lateral-most point of occipital condyle to caudal margin of palate; Figure 1), *Dasypus* (armadillo) and *Myrmecophaga* (giant anteater) had the longest ‘nose’ (measured from anteroventral margin of orbit at lacrimal foramen to anterolateral margin of nasal bone). These taxa, along with *Sus* (domestic pig) and *Tursiops* (dolphin) had the longest ‘face’ (measured from anteroventral margin of orbit at lacrimal foramen to the anterior premaxilla). At the other extreme, *Callithrix* (marmoset), *Bradypus* (sloth) and *Mustela* (weasel) showed the shortest face; *CallithrixPongo* (orangutan) and *Nomascus* (gibbon) had the shortest nose.

Those species with the longest and shortest rostra described above were not those with the largest and smallest RUNX2 QA ratios, especially in xenarthrans and afrotherians (Figure 2). Also, we did not recover a significant correlation between QA ratio and indices of rostrum size in non-carnivorans. None of our phylogenetic generalized linear models showed a significant relationship between QA ratios and relative face and nose lengths (Table 2). This was true regardless of our method of accounting for body size. In fact, closely related pairs of species that differ substantially in relative rostrum size (e.g., the anteaters *Myrmecophaga* vs. *Tamandua*; armadillos *Priodontes* vs. *Chlamyphorus*; and tenrecs *Hemicentetes* vs. *Microgale*) did not show correspondingly different QA ratios (Figure 2). For example, the extraordinarily long-faced *Myrmecophaga* had a slightly lower QA ratio (1.24) than the short-faced *Chlamyphorus* (1.53; see Figure 2).

Similar results were found for primates. The hamadryas baboon (*Papio hamadryas*) and the rhesus macaque (*Macaca mulatta*) have identical QA ratios (1.41) but the baboons have markedly longer faces (e.g. Face/Oc-Pat for baboon: 1.57, for macaque: 0.99). As is the case for the armadillo, anteater, and tenrec examples mentioned above (Figure 2), gibbons have a QA ratio similar to that of orangutans (1.35) and this also matches the ancestral reconstruction of the ratio at the great ape node. However, gibbons have much shorter faces than the great apes (e.g. Face/Oc-Pat for gibbon: 0.58, for orangutans: 0.89).

Thus, in contrast to the trend apparent among carnivorans in which long-faced species possess high QA ratios [13], this correlation does not seem to hold across placental mammals in general, and particularly not among xenarthrans or afrotherians (Table 2, Figure 2).

In order to characterize these observations further, we used the newly developed Bayesian comparative method of Lartillot and Poujol [28] (see Methods) to study the correlation between RUNX2 QA ratios and relative face/nose lengths (Table 3). As a proof of concept example, the method was first applied to the Sears et al. carnivoran dataset [13]. Using 30 *MT-CYTB* (mitochondrial cytochrome b) sequences and fossil calibrations to control for phylogeny and divergence times, the Bayesian method retrieved the positive correlation between RUNX2 QA ratio and relative facial length in Carnivora with a posterior probability of 0.95 (Table 3). However, similar analyses of our placental dataset using 41 *VWF* (von Willebrand factor) nuclear sequences and fossil calibrations to control for phylogeny and divergence times showed no significant correlation between RUNX2 QA ratio and the four relative face/nose size-controlled variables tested (Table 3). These results confirm the results of the PGLS (phylogenetic generalized least squares) analyses in demonstrating statistical independence between RUNX2 QA ratio and facial length at the global level of placentals.

Moreover, the Bayesian method also allowed the joint ancestral reconstruction of both QA and facial length ratios under the Brownian assumption (Figure 3). Figure 3a illustrates the relative homogeneity of the RUNX2 QA ratio in non-carnivoran placentals. The 95% credibility interval for the ancestral placental QA ratio is 1.14-1.80, whereas the ancestral carnivoran QA ratio is estimated at a larger value (1.57-2.63). Xenarthrans (1.01-1.65), afrotherians (1.00-1.80), and primates (1.05-1.71) showed remarkably comparable ancestral values. Also the relatively modest value (1.24-1.94) reconstructed for the Laurasiatheria ancestral node suggests that the RUNX2 QA ratio has increased in Carnivora possibly as a response to selection for increasing facial length in this clade. The Bayesian joint reconstruction of relative facial length shows a much greater variability among placentals than the QA ratio (Figure 3b). A larger heterogeneity is observed in both xenarthrans and afrotherians with relatively large ancestral facial ratios being inferred for each group. This contrasts with the homogeneity of their RUNX2 QA ratios (Figure 3a). Also, as previously noted, primates are characterized by relatively reduced facial length ratios compared to the other placental groups. This suggests that the relative facial length has been reduced in the ancestral branch leading to living primates. Overall, the comparison between the two panels of Figure 3 highlights the general lack of correlation between the RUNX2 QA ratio and relative facial length in placental mammals.

## Discussion

Our study provides the first example of using a fully integrative Bayesian method for testing a correlation between genetic and morphometric data while controlling for phylogeny, evolutionary rates, and divergence times. Our results show that the RUNX2 QA ratio is not correlated with facial length across non-carnivoran placental mammals. The RUNX2 QA tandem repeat may yet play an important role in influencing variation in face length in these mammals. However, striking changes in prognathism have occurred along several mammalian lineages without associated changes in QA ratio. Variation in QA ratio has similarly occurred without corresponding changes to facial length phenotype (Figure 2).

Another interesting result is the unusually low QA ratio for both *Choloepus* species (Linnaeus’s and Hoffman’s two-toed sloth), the only placental mammals in our sample with a QA ratio below 1 (i.e. polyA is longer than polyQ). However, this cannot be the sole factor determining the short face length of sloths as *Bradypus tridactylus* (pale-throated three-toed sloth) had a QA ratio above 1 (1.23). It should be noted, however, that the two-toed and three-toed sloths are not particularly closely related. Genetic evidence suggests that *Bradypus* and *Choloepus* evolved an arboreal lifestyle independently [29] and separated more than 20 million years ago [24]. If face length also shortened independently, then it may be that selection has acted differently upon *RUNX2* in the two-toed than in the three-toed sloth, explaining the higher QA ratio in *Bradypus*.

Some work suggests that the relationship between tandem repeats and anatomical form might not be linear [11,30]. However, with our preliminary data set of 1-10 data points per order, fitting a quadratic or exponential line seems arbitrary [31] and does not markedly improve fit. Although the nature of this study is a broad comparison across 12 orders of mammals, future work focusing on deep sampling within individual orders would be worthwhile.

In addition, Sears et al. [13] noted a weaker QA ratio in carnivorans without positively allometric growth of the face (e.g. felids). A larger sample within orders, particularly for primates [32], that accounts for variation in the degree of positive allometry across species (e.g. baboon vs. mouse lemur) may similarly reveal a stronger correlation within clades characterized by positively allometric growth of the face (e.g. papionines).

Another potential problem is the presence of intraspecific variation [32]. Although only likely to produce slight variation of the QA ratio for each species, across a phylogeny it will add to the noise within the independent contrasts analysis and reduce the signal of any potential association. Notably, examination of the *RUNX2* sequence data from the 1000 genomes project [33] indicates virtually no intraspecific variation in QA ratio among humans. This may be due to the fact that humans have undergone a recent population bottleneck and generally show relatively low levels of intraspecific variation compared to other mammals [34]. Future studies that better sample the intraspecific diversity of *RUNX2* will be able to test the extent to which the low diversity seen among humans applies across mammals.

This work represents a first step in examining the role of *RUNX2* in the evolution of facial length across placental mammals. The obvious next step is to examine possible associations between *RUNX2* tandem repeat ratios and prognathism within groups, especially those that show marked variation in facial length, such as the papionines [32,35,36]. Similarly, it would be interesting to examine variation at the other two functional domains of *RUNX2 *[15] or in the cis-regulatory regions of the *RUNX2* gene across mammals. Moreover, exciting new linkage and heritability studies of craniofacial variation in populations for which we have genetic maps [37,38] will undoubtedly yield more detailed insights on the genetics of mammalian facial morphology, including new candidate loci for cross-taxa comparisons.

## Conclusion

This study evaluates a simple molecular polymorphism (variation at the bone-growth gene *RUNX2*) that is thought to underlie functionally-important anatomical variation (relative face length). Although previous studies [11-13] demonstrated a clear, and likely causative, correlation between the QA-ratio of RUNX2 and face length in carnivorans, our analyses, controlling for phylogeny, evolutionary rates, and divergence times, show that the RUNX2 QA ratio is not generally correlated with facial length across placental mammals.

## Methods

### DNA sequence data

*RUNX2* DNA sequence data were obtained using both online genomic resources (24 species), and DNA isolation, amplification and sequencing (17 species). We retrieved DNA sequences corresponding to the poly-glutamine (polyQ) and poly-alanine (polyA) tandem repeat and flanking regions of *RUNX2* from Ensembl (releases 63 and 64; 17 species) and Genbank via BLASTN [39] (7 species) (Table 1). Notably, the QA ratio could not be reconstructed from the fragmented reads of the Neanderthal genome [40].

For the 17 species sequenced *de novo* in this study (Table 1), samples came from live animals and museum specimens, and sampling complied with institutional animal care and use protocols. Samples from live animals included feces, as well as hair collected during routine handling (e.g. for health checks). Museum specimens included skin, muscle, connective tissue, and bone. All xenarthran samples come from the Collection of Preserved Mammalian Tissues of the Institut des Sciences de l'Evolution, Montpellier [41].

We extracted DNA from approximately 0.02 to 0.05g of each sample tissue using the QIAamp DNA Mini kit (Qiagen) following the manufacturer’s instructions. PCR primer sequences are the same as those used by Sears et al. (2007). PCRs were performed using the Extensor Hi-Fidelity PCR enzyme (Thermo Scientific). An external touchdown PCR using primers Sears Ext F (5’-TTGTGATGCGTATTCCCGTA-3’) or Sears Int F (5’ATCCGAGCACCAGCCGGCGGCGCTTCAG-5’) with Sears Ext R (5’-ACSGAGCACAGGAAGTTGGG-3’) was performed on approximately 100ng of template DNA with the following cycling conditions: 95°C 2mins, 15x (95°C 30s, 61.3 (down 0.5°C per cycle) 30s, 72°C 50s), 20x (95°C 30s, 54.3°C 30s, 72°C 50s), 72°C 5 mins final extension. The product of the external PCR was then diluted to a 1/10 concentration and 0.5μl was added as template for the internal PCR. This nested PCR using primers Sears Int F and Sears Int R (5’-GTGGTCVGCGATGATCTCSAC-3’) was run with the following cycling condition: 94°C 3 mins, 40x (94°C 30s, 62°C 45s, 72°C 45s), 72°C 5 mins final extension. The product of this second reaction was run out on a 1.5% agarose gel containing ethidium bromide. When an amplicon of roughly 300bp in size was visible under UV light, the DNA was extracted, purified (QIAquick PCR purification kit, Qiagen) and sequenced (Big Dye, version 1.3).

The *RUNX2* DNA sequences were analyzed and translated using sequence alignment and editing software (Lasergene, DNASTAR, BioEdit, ClustalW) and the QA ratios were calculated from the translated sequences. Sequences of sufficient length (> 100bp) have been deposited in GenBank (accession numbers: JQ405327 - JQ405335).

### Morphological measurements

We quantified facial length for the 41 placental mammal species listed above by measuring adult crania from museum collections in Cambridge (UMZC, University Museum of Zoology, Cambridge), Berlin (ZMB, Zoologisches Museum Berlin), New Haven (YPM, Yale Peabody Museum), Washington DC (USNM, Smithsonian Institution, National Museum of Natural History) and New York (AMNH, American Museum of Natural History). Specimen numbers for each species are listed in the Supplementary Material ( Additional File 2). We took four measurements of each cranium with digital calipers (Face, Nose, CBL, Oc-Pat; Figure 2) based on the landmarks described by Sears et al. [13]. We measured both total facial length (Face), measured from the anteroventral margin of the orbit at the lacrimal foramen to the anterior margin of the premaxilla, and nasal length (Nose), measured from the anteroventral margin of the orbit at the lacrimal foramen to the anterolateral margin of the nasal bone. We also took two proxy measures of body size: Cranial base length (Oc-Pat), i.e., the distance from lateral-most point of the occipital condyle to the caudal margin of the palate, and condylobasal or total cranial length (CBL), i.e., the distance from the lateral-most point of the occipital condyle to the anterior-most premaxilla, as illustrated in Figure 2. These measures of skull length are positively and significantly correlated with body mass across a wide range of mammals [42,43]. For most species that exhibit sexual dimorphism and for which information on sex was available in museum collections, we measured at least two males and two females. Although the males and females of markedly dimorphic species (e.g. chimpanzee, gorilla, baboon) differed by as much as 20% for the absolute values of nose and face length, the relative measurements within species were similar, especially using total cranial length (CBL) as a proxy for body size (Table 1). Species averages for each measurement are given in the supplementary material ( Additional File 3).

We standardized the two absolute measures of facial length (Face and Nose) by dividing each by body size proxies: cranial base (Oc-Pat) and condylobasal length (CBL). This gave us two relative values for each measurement.

Face length measures for humans seem longer than expected because on the human cranium the occipital condyles are closer to the palate, as the foramen magnum points ventrally. Therefore, ratios with CBL in the denominator may appear apelike due to the proximity of foramen magnum-palate, not due to a long face.

### Comparative analyses

Interspecific datasets cannot be analyzed using traditional statistical methods because of sample non-independence due to shared evolutionary history [44]. Phylogenetically independent contrasts [45] have been commonly used in comparative studies, yet this approach assumes that trait variation perfectly follows a Brownian motion pattern across the phylogenetic tree (i.e. traits exhibit strong phylogenetic signal).

A more recent approach, phylogenetic generalized least squares (PGLS) [46], is more powerful [47]. This method includes an extra parameter, lambda, which varies continuously from zero to one and is optimized by maximum likelihood. A lambda value of zero indicates that phylogeny has no importance in the model, and is in fact identical to a model that does not account for the evolutionary history of species. A value of one indicates that the model’s error structure follows Brownian motion perfectly. In practice, many models exhibit a lambda value somewhere between zero and one. This and related methods are becoming increasingly popular in comparative biology with the greater usability of advanced statistical software [48-50].

We conducted PGLS analyses using the caper package for R [51,52]. Before analysis we natural log transformed the variables to better meet the assumptions of parametric statistics [53]. We constructed a phylogeny, including branch lengths as represented by estimated divergence times, from a number of sources [26,27,54-56]. These studies did not use the same data to calibrate their trees. Therefore, we used the published divergence times for the terminal taxa but had to adjust the divergence times for some of the deeper branches. This was necessary because the distance between each terminal taxon and the root of the tree needs to be consistent (i.e. the tree should be ultrametric). In addition, we examined how sensitive our results were to our choice of phylogeny by conducting a second set of analyses using the mammal supertree presented in Bininda-Emonds [57,58]. These models produced qualitatively similar results to our initial analyses. We examined our results for outliers by visually inspecting Q-Q plots and fitted vs. observed value plots. We also defined outliers as data points that exhibited standardized phylogenetic residuals greater than three or less than negative three [53].

We also used a recently developed Bayesian comparative method implemented in the CoEvol program [28]. This integrative approach uses a Bayesian framework that allows joint reconstruction of variations in molecular evolutionary rates, divergence times, and continuous variables. These parameters are modeled as a multivariate Brownian diffusion process along the branches of the phylogenetic tree while taking their covariance into account. We applied this method to evaluate the correlation between the RUNX2 QA ratio and four size-controlled face/nose length variables while controlling for phylogeny, evolutionary rates and divergence times.

We first applied this Bayesian method to the Sears et al. dataset [13]. Since this is the only marker for which the 30 species measured by Sears et al. [13] have been sequenced, we used the mitochondrial *MT-CYTB* gene to control for phylogeny along with the topology and the eight fossil calibrations proposed by Eizirik et al. [59]. We then applied the same methodology to our placental dataset. In this case however, we used *VWF* exon 28 nuclear sequences to control for phylogeny, because it is the only gene currently available for the 41 species of our sample. We also used the 20 fossil calibrations proposed by Benton et al. [60] that were compatible with our taxon sampling. In both cases, CoEvol was run for 30,000 MCMC cycles sampling parameters every 10 cycles. The first 500 samples were discarded as the burnin and posterior averages were estimated on the remaining 2500 points. The CoEvol datasets have been deposited in the Dryad Repository [61].

## Abbreviations

RUNX2, Runt-related transcription factor 2; QA ratio, ratio of glutamines to alanines; CBL, distance from lateral-most point of occipital condyle to anterior premaxilla; Oc-Pat, distance from lateral-most point of occipital condyle to caudal margin of palate; NOSE, anteroventral margin of orbit at lacrimal foramen to anterolateral margin of nasal bone; FACE, anteroventral margin of orbit at lacrimal foramen to anterior premaxilla; MT-CYTB, mitochondrial cytochrome b; VWF, von Willebrand factor.

## Competing interests

The authors have no competing interests.

## Author contributions

Conceived and designed the project: MAP, BJB, RJA, NIM. Collected and analyzed data: MAP, BJB, JMK, VW, SGBC, FD, RJA. Wrote the paper: MAP, BJB, RJA, JMK, FD. Edited the manuscript: all authors. All authors read and approved the final manuscript.

## Supplementary Material

Additional file 1 **Amino acid alignment of RUNX2 showing the QA region with some flanking sequence.** An asterisk indicates that this sequence was identical in more than one individual. Click here for file

Additional file 2 Source and specimens IDs for skulls measured for each species. Click here for file

Additional file 3 Skull measurements for each species (in cm). Click here for file
